# Parallel genetic adaptation across environments differing in mode of growth or resource availability

**DOI:** 10.1002/evl3.75

**Published:** 2018-08-04

**Authors:** Caroline B. Turner, Christopher W. Marshall, Vaughn S. Cooper

**Affiliations:** ^1^ Microbiology and Molecular Genetics University of Pittsburgh Pittsburgh Pennsylvania

**Keywords:** Biofilm, *Burkholderia*, experimental evolution, nutrient limitation, parallel evolution, whole‐population sequencing

## Abstract

Evolution experiments have demonstrated high levels of genetic parallelism between populations evolving in identical environments. However, natural populations evolve in complex environments that can vary in many ways, likely sharing some characteristics but not others. Here, we ask whether shared selection pressures drive parallel evolution across distinct environments. We addressed this question in experimentally evolved populations founded from a clone of the bacterium *Burkholderia cenocepacia*. These populations evolved for 90 days (approximately 600 generations) under all combinations of high or low carbon availability and selection for either planktonic or biofilm modes of growth. Populations that evolved in environments with shared selection pressures (either level of carbon availability or mode of growth) were more genetically similar to each other than populations from environments that shared neither characteristic. However, not all shared selection pressures led to parallel evolution. Genetic parallelism between low‐carbon biofilm and low‐carbon planktonic populations was very low despite shared selection for growth under low‐carbon conditions, suggesting that evolution in low‐carbon environments may generate stronger trade‐offs between biofilm and planktonic modes of growth. For all environments, a population's fitness in a particular environment was positively correlated with the genetic similarity between that population and the populations that evolved in that particular environment. Although genetic similarity was low between low‐carbon environments, overall, evolution in similar environments led to higher levels of genetic parallelism and that genetic parallelism, in turn, was correlated with fitness in a particular environment.

**Impact Summary**

The roles of chance and determinism in the process of evolution are a perpetually fascinating question. As evolutionary biologist Stephen Jay Gould famously asked, what would happen if we “replayed the tape of life”? If we went back in time and replayed evolution from the exact same starting point, would we see the same outcome because organisms are evolving in the same environments with the same selection pressures? Or would we see different outcomes because different random mutations occur, leading evolutionary history in a different direction? In this article, we ask a related but slightly different question. What happens when organisms evolve in environments that are not identical, but do share some particular environmental characteristic? We evolved replicate populations of bacteria in different environments that varied in two major characteristics. We showed that bacteria evolved in environments that shared one of the characteristics were generally more similar to each other than bacteria evolved in environments that did not share either of the two characteristics. Specifically, the bacteria evolved in environments with shared characteristics also shared mutations in many of the same genes in most, but not all cases. Our results indicate that repeatability in evolution can be observed, even when the evolutionary environment differs.

The repeatability or predictability of evolution is a central question in evolutionary biology, most famously posed by Stephen Jay Gould when he asked what would happen if we “replay the tape of life” (Gould 1990). Results from evolution experiments indicate that although evolution is not identical in replicate populations, there is an important degree of predictability to evolution (Lässig et al. 2017). Genomic analysis of populations experimentally evolved under controlled, identical conditions has consistently revealed parallelism in which mutations in certain genes are repeatedly selected (Wichman et al. 1999; Toprak et al. 2012; Tenaillon et al. 2016; Venkataram et al. 2016). However, natural environments are rarely identical, raising the question of how environmental differences affect genetic parallelism. As would be expected, populations evolved in different environments are generally less genetically similar than populations evolved in identical environments (Bailey et al. 2015; Deatherage et al. 2017). Less clear however is whether genetic similarity is higher between environments with a common selection pressure than between environments lacking that commonality.

All else being equal, we might assume that mutations that confer adaptive benefits for a particular selection pressure in one environment would also be beneficial in another environment with the same selection pressure. However, several factors could reduce parallelism between these similar, but distinct environments. Antagonistic pleiotropy could occur, such that mutations that are beneficial in adapting to the shared selective pressure in one environment are detrimental in another environment (MacLean et al. 2004; Flynn et al. 2013). Furthermore, even if the same mutations are beneficial in both environments, the distribution of their fitness effects might differ, such that the mutations that are most beneficial differ between environments (Deatherage et al. 2017). This difference in fitness effects could lead to reduced parallelism as mutations with a larger fitness benefit will spread more rapidly, particularly in clonal organisms where beneficial mutations occurring on different backgrounds cannot recombine in the absence of horizontal gene transfer (Gerrish and Lenski 1998; Levy et al. 2015).

Observational studies of local adaptation have produced mixed results as to whether genetic parallelism underlies how populations adapt to a particular selective factor, even when other aspects of their environment vary (Kawecki and Ebert 2004). Supporting the idea of parallelism across environments sharing a common parameter, the evolution of heavy metal and pesticide tolerance across different plant populations can often be traced to the same loci (Schat et al. 1996; ffrench‐Constant et al. 1998). In a meta‐analysis, Conte et al. (2012) found genetic parallelism in 30–50% of studied cases where similar phenotypes evolved in multiple populations. Interestingly, the evolution of quinolone resistance among *Pseudomonas aeruginosa* populations causing lung infections is most often caused by mutations in DNA gyrase A, regardless of differences in the evolutionary environment due to different patient types (Wong and Kassen 2011). However, other resistance mutations occurred disproportionately either in patients with cystic fibrosis or in patients without cystic fibrosis, indicating that some, but not all, mutations were shared in adaptation to a shared selection pressure in distinct environments. The drawback of these observational studies is that the degree of variation among habitats cannot be controlled, or even measured, given the indeterminately large number of potential environmental variables involved. By systematically varying a few environmental characteristics in a laboratory experiment, and conducting whole‐genome sequencing, we can directly measure the degree of parallelism between populations evolved in environments with and without different shared selection pressures.

There is little experimental evidence testing whether populations evolved in environments with more shared characteristics exhibit higher levels of genetic similarity. Unlike in natural evolution, laboratory evolution experiments most commonly vary a single selective factor such as resource availability (Gresham et al. 2008), temperature (Bennett and Lenski 2007), or presence of a coevolving predator (Lennon and Martiny 2008; Meyer et al. 2012). Studies manipulating multiple factors are much less common (Bohannan and Lenski 1997; Wong et al. 2012). Furthermore, whole‐genome sequencing of these experiments has only become feasible relatively recently. Wong et al. (2012) is the only evolution experiment we are aware of where multiple environmental characteristics were varied in a factorial design and evolved strains were sequenced. Although Wong et al. did not directly test the effect of environmental similarity, they did observe mutations in common between environments with shared characteristics.

Observations of genetic parallelism across nonidentical environments raise a further question: whether the degree of genetic parallelism between populations is predictive of fitness in reciprocal environments. Indeed, local adaptation studies suggest that populations that evolved in more similar environments have higher reciprocal fitness (Becker et al. 2006; Raabová et al. 2007). If populations in a particular focal environment repeatedly accumulate parallel mutations in the same genes, then those mutations are highly likely to be beneficial in that environment. Therefore, we would expect that populations evolved in other environments that also have mutations in those genes should be more fit in the focal environment than populations that lack those mutations. However, there are several reasons why this might not be the case. First, genetically dissimilar organisms might have equivalently high fitness by carrying out similar functions using mutations in a different set of genes (Wittkopp et al. 2003; Yoon and Baum 2004). Second, epistatic interactions could cause parallel mutations to have different outcomes due to other differences in the genome (Kvitek and Sherlock 2011; Wang et al. 2013). Additionally, although genetic parallelism is usually measured and observed at the level of genes (or higher) rather than at the level of individual nucleotides (e.g., Kvitek and Sherlock 2011; Gerstein et al. 2012; Deatherage et al. 2017), different evolved mutations within a gene may have different phenotypic effects (Applebee et al. 2008; Rodríguez‐Verdugo et al. 2013). Such cases would weaken the correlation between genetic parallelism and reciprocal fitness.

To examine the relationships among environmental similarity, genetic parallelism, and reciprocal fitness, we evolved the bacterium *Burkholderia cenocepacia* under a factorial design varying two important environmental characteristics: nutrient level and mode of growth. The availability of resources is a key environmental variable affecting all organisms. Many organisms are adapted to be better competitors in low‐resource environments, others in high‐resource environments. For bacteria specifically, biofilm growth on surfaces and planktonic growth suspended in liquid require different responses. Bacteria growing on surfaces secrete a variety of molecules to improve attachment and grow in spatially structured habitats with close physical proximity to other cells (Hall‐Stoodley et al. 2004). By contrast, planktonic selection primarily favors organisms that can take up nutrients the fastest or draw the availability of nutrients down to the lowest level (Tilman 1982; Vasi et al. 1994).

After 90 days of evolution, we first tested whether genetic parallelism is higher between environments that share a selection pressure, either nutrient level or mode of growth. Overall, genetic parallelism was higher in more similar environments. However, we also observed a lack of parallelism specifically between populations selected for biofilm or planktonic growth under low‐nutrient conditions. The relationship between genetic parallelism and fitness in reciprocal environments was more consistent, showing a positive correlation in all of the evolutionary environments.

## Methods

### EVOLUTION EXPERIMENT

**Figure 1:**
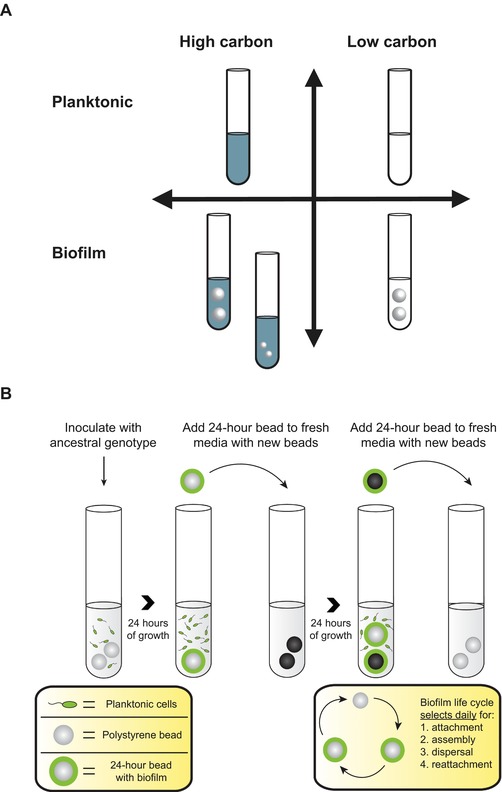
Experimental Design. (A) Six replicate populations were evolved under each of five treatments. Two treatments were evolved under high‐carbon, biofilm conditions, one with large beads, and one with small beads. The small‐bead treatment had a comparable bottleneck population size to the low‐carbon treatments and thus acted as a control for population size. (B) In biofilm‐selected populations, a bead, instead of liquid, is transferred to a new tube daily.

We propagated 30 populations of *B. cenocepacia* for 90 days in media with high or low carbon and selected for either planktonic or biofilm growth (Fig. 1). We used a fully factorial design with all four combinations of carbon availability and selection on mode of growth. In addition, as a control for the differences in population size between high‐carbon and low‐carbon treatments, we implemented a fifth treatment with biofilm selection under high‐carbon conditions, but with a bottleneck population size comparable to the low‐carbon populations (Fig. S1). Six replicate populations evolved in each treatment.

All populations were founded from a clone of *B. cenocepacia* HI2424, originally isolated from agricultural soil (LiPuma et al. 2002; Poltak and Cooper 2011). Half were founded from a single Lac^+^ clone and the rest from a single Lac^–^ clone (Poltak and Cooper 2011). Other than the lactose marker, the two clones were genetically identical, as confirmed by genomic sequencing. All populations were evolved in test tubes with 5 mL of M9 minimal media (0.37 mM CaCl_2_, 8.7 mM MgSO4, 42.2 mM Na_2_HPO_4_, 22 mM KH_2_PO_4_, 21.7 mM NaCl, and 18.7 mM NH_4_Cl). The high‐carbon medium contained 8.7 g/L galactose and the low‐carbon medium contained 0.26 g/L galactose as the sole carbon source. Galactose concentration was limiting to planktonic population size in both high‐ and low‐carbon media.

Bacteria were transferred to fresh media every 24 h. For planktonic populations, we transferred 50 μL of liquid to 5 mL fresh media. For biofilm populations, we transferred a polystyrene bead (Polysciences, Inc., Warrington, PA) to fresh media containing two sterile beads. Each day we alternated between black and white marked beads, such that the bacteria on the transferred bead had always been growing for 24 h (Fig. 1B). This process of transferring beads selects for bacteria that attach to the bead surface, then disperse and attach to a new bead after transfer. For the standard high‐ and low‐carbon biofilm treatments, we used a larger bead with a diameter of 6 mm. To control for the effects of population size, one treatment with high‐carbon media was transferred with a 3 mm diameter bead, giving a bottleneck population size similar to that of the low‐carbon treatments (Fig. S1). All populations were incubated at 37°C in a roller drum rotating at 30 rpm.

Every 15 transfers, we froze a sample of each bacterial population. For planktonic populations, we froze 1 mL of liquid culture with 8% DMSO as a cryoprotectant in a −80°F freezer. For biofilm populations, we suspended the bacteria from one bead in 1 mL sterile galactose‐supplemented M9 minimal media with 8% DMSO.

### GENOME SEQUENCING

We sequenced whole‐population samples of each of the populations following 90 days of evolution. Each sample was revived from a freezer stock in tryptic soy broth, and then maintained for two days under evolutionary conditions. Genomic DNA was isolated using a Qiagen DNeasy Blood and Tissue kit (Qiagen, Hilden, Germany). The sequencing library was prepared using the Illumina Nextera kit (Illumina Inc., San Diego, CA) with modifications following Baym et al. (2015). All samples were sequenced to at least 115‐fold average coverage using an Illumina NextSeq 500. Following sequencing, we trimmed the samples with Trimmomatic (version 0.36, Bolger et al. 2014) and called mutations by comparing evolved populations to the ancestral genome, *B. cenocepacia* HI2424 (GCF_000203955.1), using breseq (version 0.28, Deatherage and Barrick 2014) with the standard settings for identifying polymorphic mutations. These settings identify only mutations that reach a frequency of 0.05 in the population and that occur on at least two reads from each strand. Mutation calls were manually curated to remove false positives due to misaligned reads and to consolidate sequential single base pair insertions or deletions into a single multiple base pair insertion or deletion. To test whether our choice of minimum frequency affected our results, we also ran our analyses with only those mutations that reached at least 0.1 frequency in the population.

### GENETIC SIMILARITY

When analyzing genetic similarity, we included only mutations that affected a single gene. Following the procedure of Deatherage et al. (2017), we excluded synonymous mutations, mutations that affected multiple genes, and intergenic mutations that were not within 150 base pairs upstream of a gene. This approach meant that we included only mutations whose effects could unambiguously be assigned to a specific gene.

The Bray–Curtis metric is frequently used in ecology as a measure of similarity of species composition between communities. Analogously, we here apply Bray–Curtis as a measure of the genetic similarities between populations. We calculated the Bray–Curtis similarity between each pair of evolved populations, *i* and *j*, where *n_ig_* is the frequency of mutations in gene *g* in population *i* for all genes in the genome:

$$\;BC_{ij}=\;1-\;\frac{\sum_{g\;=\;1}^{G}\left|n_{ig}-n_{jg}\right|}{\sum_{g\;=\;1}^{G}\left(n_{ig}+n_{jg}\right)}.$$

We applied the metric at the level of the gene, meaning that mutations at different nucleotides within a single gene and upstream region were summed together. For genes without mutations in either population (the majority of genes in the genome), *n_ig_* and *n_jg_* are both zero, thus these genes do not affect the Bray–Curtis similarity. Bray–Curtis similarity incorporates both the identity of the genes mutated in each population and the frequency of those mutations. It ranges from 0, when two populations do not have mutations in any of the same genes, to 1, when two populations have mutations in all the same genes at identical frequencies. Because there may be frequency‐dependent interactions between mutations in our system, particularly in biofilm environments (Traverse et al. 2013; Ellis et al. 2015), it is valuable to use a metric that incorporates the frequency of mutations in genes, and not just presence/absence. We calculated the mean within‐treatment similarity (BC_within_) as the average of the Bray–Curtis similarity for all pairwise combinations of replicate populations that shared a treatment. Similarly, the mean between‐treatment similarity (BC_between_) is the average of the Bray–Curtis similarity for all pairwise combinations of populations that evolved under different treatments.

**Figure 2:**
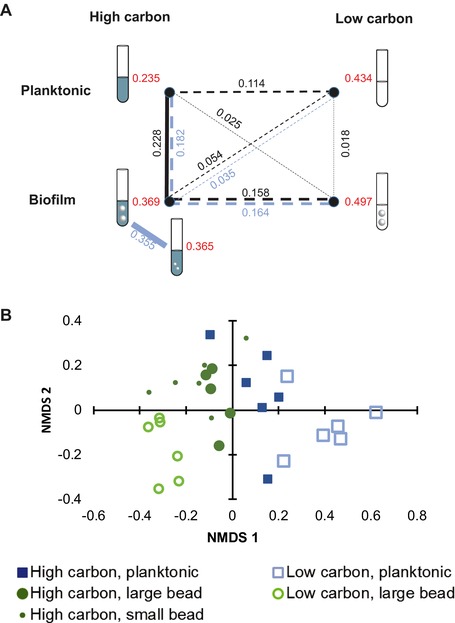
(A) Bray–Curtis similarity was significantly higher within (red) than between (black/blue) treatments. Dashed lines indicate pairs of treatments for which similarity between treatments was significantly lower than similarity within treatments. Between‐treatment similarity was significantly higher for pairs of treatments with a shared environmental variable (edges of rectangle) than for pairs that did not share an environmental variable (diagonals of rectangle). Blue lines and numbers indicate comparisons involving the high‐carbon, biofilm treatment with small beads. Black lines are used for all other treatment pairs. (B) Nonmetric multidimensional scaling plot of population mutation profiles shows a significant effect of carbon concentration (permutation analysis of variance, *P* = 0.001), mode of growth (*P* = 0.001), and the interaction between the two (*P* = 0.004).

We tested whether the populations were more genetically similar within treatments than between treatments using a randomization approach (Deatherage et al. 2017, see Text S1 for details). Similarly, we determined whether the genetic similarity between treatment pairs was higher between treatments that shared an environmental trait (either carbon level or mode of growth, edges of the rectangle in Fig. 2A) than between treatments that did not share either trait (diagonals of the rectangle in Fig. 2A). We determined significance with a cutoff value of *P* < 0.05.

### FITNESS MEASUREMENTS

We measured the fitness of each evolved population compared to the ancestor in each of the five evolutionary environments. We revived and acclimated freezer stocks as described above and then added equal volumes of evolved populations and the oppositely marked (lac^+^/lac^–^) ancestor to a competition tube. We plated dilutions of the initial population onto tryptic soy agar supplemented with X‐gal, which allowed us to enumerate the population size of each competitor based on colony color. After 24 h of incubation, we transferred either a bead (for biofilm environments) or 50 μL of liquid (for planktonic environments) to a new test tube. At 48 h, we again plated onto X‐gal plates. Fitness was calculated as the difference between the Malthusian parameters for the two competitors, that is, selection rate (day^−1^) = (ln(evolved_d = 0_/evolved_d = 2_) − ln(ancestral_d = 0_/ancestral_d = 2_))/2. A selection rate of zero indicates that the two competitors are equally fit, whereas a selection rate above or below zero indicates that the evolved strain is more or less fit, respectively, compared to the ancestral strain in that environment.

### GENETIC SIMILARITY VERSUS RECIPROCAL FITNESS

Our next goal was to test whether fitness of a population in a particular environment could be predicted by the level of genetic similarity between that population and the populations that evolved in a given environment. We measured the fitness of all populations in all five environments as described above. To calculate the genetic similarity of a population to the populations evolved in a particular environment, we calculated the mean Bray–Curtis similarity between that population and each of the populations evolved in that environment. For example, for population 1 from the high‐carbon, planktonic environment, genetic similarity to the low‐carbon, planktonic environment was calculated as the mean of the Bray–Curtis similarity of population 1 to each of the six populations that evolved in the low‐carbon, planktonic environment. For populations native to a given environment, we used the mean similarity to the other populations in the same treatment, excluding its self‐similarity (which is, by definition, equal to 1). We then tested whether fitness in a given environment was correlated to genetic similarity. Because we had no reason to expect a linear relationship between similarity and fitness, we used the nonparametric Kendall's rank correlation coefficient for this analysis. All data analysis was done using R (version 3.2.2) with the vegan package used for Bray–Curtis similarity calculations (Oksanen et al. 2107).

## Results

### EXPERIMENTAL EVOLUTION

Following evolution in environments varying in carbon availability and mode of growth, populations showed very large and significant improvements in fitness in their evolutionary environments compared to the ancestral clone (Fig. S2). Selection rate averaged 2–3/day in biofilm conditions and 1.8/day in planktonic conditions. Populations evolved under selection for biofilm formation produced three to times as much biofilm as the ancestral clones, but planktonic‐evolved populations did not differ significantly from the ancestor in biofilm production (Fig. S3). We excluded one high‐carbon, large‐bead replicate population from all analyses due to cross‐contamination.

### GENOMIC SEQUENCING

We sequenced whole‐population samples from 29 populations following 90 days of evolution to a coverage depth of 220 ± 46 (mean ± SD) coverage. Across all populations, after removing false positives, we observed 432 mutations, an average of 14.9 mutations per population. For calculations of genetic similarity, we included only mutations that could be assigned to a single protein. Therefore, we excluded 55 synonymous mutations and 25 intergenic mutations that were not within the likely promoter region, 150 bp upstream of a gene (Deatherage et al. 2017). The remaining 352 mutations were included in the analyses of genetic similarity. Of these 352 mutations, 51 spread to fixation in a population, whereas the remaining 301 mutations were present at intermediate frequencies. The number of mutations did not differ significantly by treatment (Kruskal–Wallis test, *P* = 0.052), though there was a trend of lower numbers of mutations in the low‐carbon treatments. In addition to the mutations identified by breseq, it is worth noting that there was also evidence for a deletion of a region containing 95 genes, also observed in a prior experiment (Traverse et al. 2013), in several of the populations. The fitness effect of this deletion is likely driven by deletion of the gene *rpfR*, for which we also observed many point mutations in this experiment. However, because the deletion cannot be assigned to a single gene, we did not include it in our analysis.

### GENETIC SIMILARITY VERSUS ENVIRONMENTAL SIMILARITY

Overall, genetic similarity was greater within treatments than between treatments (Fig. 2A, randomization test, *P* < 10^−5^). The overall within‐treatment similarity (Bray–Curtis similarity among populations within a treatment, BC_within_) was 0.373, whereas the mean similarity between treatments (BC_between_) was 0.129. All pairs of treatments differed significantly from each other with two exceptions (Fig. 2A; Table S1). First, the similarity between populations evolved in the two high‐carbon, biofilm environments (large bead and small bead) was not significantly higher within treatments than between treatments. This result suggests that population size at transfer (which was initially ∼9 × 10^7^ in the large‐bead treatment, but ∼3 × 10^7^ in the small‐bead treatment, Fig. S1) did not drive differences in the identity, frequency, or probability of fixation of mutations. The other exception was between the high‐carbon, large‐bead, and the high‐carbon, planktonic treatments. This surprising result was driven both by the presence of shared mutations between the treatments and the relatively low within‐treatment similarity in the high‐carbon, planktonic populations.

Further, populations evolved in environments with the same carbon concentration or under the same mode of growth exhibited more genetic similarity than populations evolved in environments that did not share these traits (randomization test, *P* < 10^−5^). A notable exception was the pairing between the two low‐carbon environments. Although these environments had a shared selection pressure, the mean Bray–Curtis similarity between them was 0.018, comparable to or even lower than the similarity between pairs of environments that did not share characteristics (Fig. 2A). To test whether these results were influenced by our choice to use a 0.05 frequency cutoff for mutations, we also analyzed Bray–Curtis similarity for only those mutations that reached a frequency of at least 0.1 in the population. We also analyzed similarity by Euclidean distance and Jaccard similarity with only presence/absence of genes and by Euclidean distance. All results were the same as for Bray–Curtis with a 0.10 frequency cutoff, except that for the Euclidean distance metric, the low carbon treatments did not have low levels of similarity with each other (Table S4).

The degree of differentiation between populations evolved in different environments can also be seen in a nonmetric multidimensional scaling plot (Fig. 2B). Carbon concentration (*P* = 0.001), mode of growth (*P* = 0.001), and the interaction between the two (*P* = 0.004) all had a significant effect on genetic distance. Consistent with the results from the Bray–Curtis analysis, low‐carbon biofilm and low‐carbon planktonic populations were most distant from each other, whereas high‐carbon populations differed less across treatments.

### GENETIC SIMILARITY VERSUS RECIPROCAL FITNESS

We next considered whether the degree of genetic similarity predicts the fitness of populations grown in novel environments, following the rationale that populations that are genetically similar may share adaptations to common conditions. For example, because populations evolved in the two planktonic environments shared mutations in some of the same genes, we might expect that populations evolved in the high‐carbon, planktonic environment would be better adapted in the low‐carbon, planktonic environment than populations evolved in the high‐carbon, biofilm environments. Alternatively, other differences between the populations might outweigh the effects of similarity between the populations.

**Figure 3:**
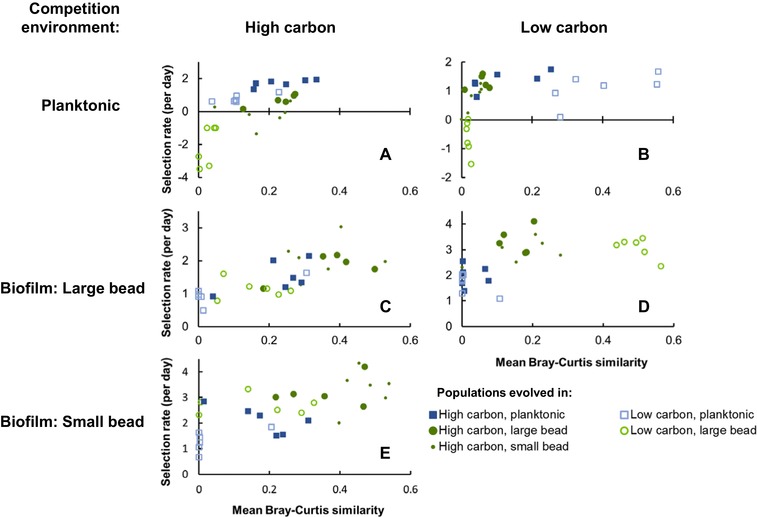
Correlation between genetic similarity and fitness (selection rate, per day) in a given environment (Kendall's rank correlation test, *P* < 0.001 in all cases). Mean Bray–Curtis similarity is calculated as the average pairwise similarity between a given population and all populations that evolved in the tested environment. Selection rate is measured by competing evolved populations against oppositely marked ancestral clones in the evolutionary environment indicated above and to the left of the plot. A selection rate of zero indicates equal fitness between the two competitors. Note that scale of the *y*‐axis differs between plots.

Overall, the degree of genetic similarity to populations evolved in a given environment was a significant predictor of fitness in that environment (Fig. 3; Table S2; Kendall's rank correlation test *P* < 0.001 in all cases). To test if this relationship was driven solely by higher fitness of the populations native to a particular environment, we also calculated the correlation between fitness and environmental similarity for only nonnative populations in each environment. In all cases, the significant positive correlation between fitness and genetic similarity remained (Kendall's rank correlation test, *p* < 0.01 in all cases; Table S2).

These results also suggest the possibility that genetic similarity may be a better predictor of fitness than shared environmental characteristics. Because genetic and environmental similarity are correlated and only five different environments were studied, our experiment has limited power to identify which better predicts fitness in alternative environments and we have not conducted a formal statistical test. However, comparisons between the populations evolved in low‐carbon environments are suggestive. These populations share a history of evolving in low‐carbon conditions, but planktonic‐evolved lines showed very little genetic similarity to biofilm‐evolved lines. Reciprocal fitness between low‐carbon environments was very low (Fig. 3B and D, unfilled symbols), indicating that in this case the lack of genetic similarity better predicted fitness than a common environmental variable.

### IDENTITY OF GENES WITH PARALLEL MUTATIONS

**Figure 4:**
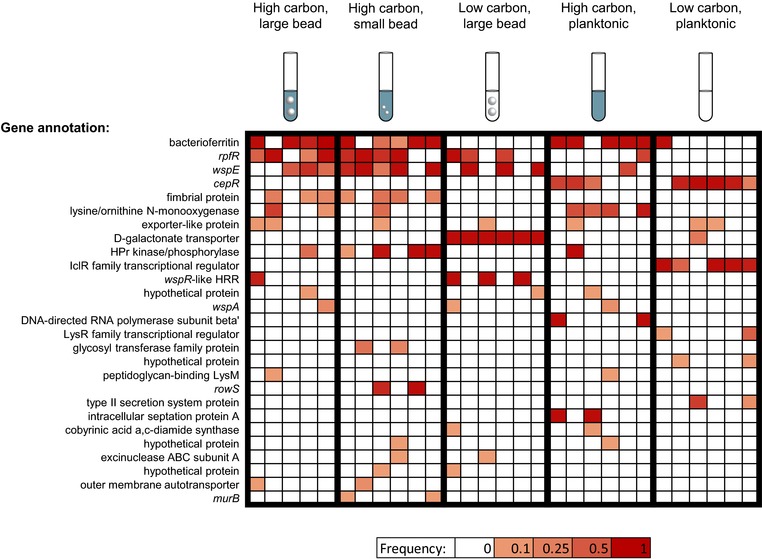
Genes in which mutations were observed in more than one population. Each column represents one replicate population. Color indicates the total frequency of mutations in that gene in that population.

We can infer that selection acted upon genes (or gene products) in which mutations reached high frequency repeatedly in independent populations. In fact, the population genetic conditions in this experiment, with *N*_e_ > 3 × 10^7^ and extremely strong selection (Fig. S2), were such that almost all mutations that rose to detectable frequency were under positive selection (Desai and Fisher 2007; Good et al. 2012) or were hitchhiking with a mutation under positive selection. Twenty‐seven genes were mutated in at least two evolved populations (Fig. 4) and 10 genes were significantly associated with changes in fitness in different environments (Fig. S4). The three most commonly mutated genes in this experiment, bacterioferritin, *rpfR* (also denoted *yciR*), and *wspE*, have also been mutated previously in other experiments with *B. cenocepacia* evolving in the bead biofilm model (Traverse et al. 2013; Cooper et al. 2014; O'Rourke et al. 2015). In this experiment as in previous experiments, *rpfR* and *wspE* mutations were associated with populations selected for biofilm formation. Both *rpfR* and *wspE* are part of signaling pathways that regulate biofilm production (Goymer et al. 2006; Deng et al. 2012; Traverse et al. 2013). Another regulator relevant to biofilm production, the quorum‐sensing regulator *cepR* (Huber et al. 2001) was repeatedly disrupted in planktonic populations evolved in either high or low carbon. Bacterioferritin mutations were strongly associated with high‐carbon environments. Because bacterioferritin is an iron storage protein and iron is not supplemented in the medium, it is likely that the high‐carbon populations but not low‐carbon populations experienced iron limitation or colimitation at some point in their evolutionary history (Traverse et al. 2013). In some cases, parallelism was restricted to a single treatment, such as the galactonate transporter (Winsor et al. 2008) in which mutations are fixed in all six low‐carbon, biofilm populations, but not in any other population (though a mutation does occur at intermediate frequency in one low‐carbon planktonic population). These mutations appear to modify the transmembrane helices of this transporter and could make it more permissive for galactose, the sole carbon source supplied in the media. Consistent with the lack of overall genetic similarity between low‐carbon biofilm and low‐carbon planktonic populations, we observe no gene that was mutated in parallel across low‐carbon environments (Fig. 4).

We have focused in this article on parallelism at the gene level, however parallelism can also occur at other levels (Tenaillon et al. 2012). We observed extensive parallelism at the level of individual nucleotides. In the most striking example, all 15 populations with mutations associated with the bacterioferritin (*bfr*) gene had a mutation 61 bp upstream of the gene at some frequency in the population. A mutation at this nucleotide also rose to high frequency in a previous evolution experiment (Traverse et al. 2013), where it was shown to increase transcription levels of the *bfr* gene. The bacterial promoter prediction software BPROM (Solovyev and Salamov 2011) estimates that this mutation nearly doubles the affinity of the ‐35 promoter box for σ70 promoters. These results suggest a strong fitness benefit to mutations at this specific nucleotide. (Note that these mutations are unlikely to have simply been present at low frequency in the ancestral stock, given that they arose in both the lac^+^ and lac^–^ populations as well as in the previous evolution experiment.) Parallelism at the nucleotide level also occurred in *rpfR*, *wspE*, and the galactonate transporter (see Table S3 for a detailed list of mutations).

Our metric of genetic similarity also does not account for cases of parallelism where mutations repeatedly spread in multiple genes within a pathway. For example, our analysis considers mutations in the genes *wspE*, *wspA*, and *wspR*‐like hybrid response regulator (HRR) to be distinct, although they produce proteins in the same signaling complex (Cooper et al. 2014). Furthermore, mutations in *wspE* and *wspR*‐like HRR occur in a disjoint set of populations, suggesting that mutations in the two genes might be serving similar functions but the occurrence of one precludes the other, as our model of this signaling complex predicts (Cooper et al. 2014; O'Rourke et al. 2015). When mutations were grouped by operon, similarity within treatments was somewhat higher, but the overall pattern of similarity between treatments remained the same (Table S4).

## Discussion

In our experiments, we asked two simple questions. (1) Do populations evolving in more similar environments exhibit more genetic similarity? (2) Are populations with more genetic similarity more fit in each other's evolutionary environment than populations with less genetic similarity? We demonstrated that the overall answer was yes to both questions, although not in all cases. Our results suggest that populations adapting to common selection pressures, such as environmental change, may adapt via a common genetic toolkit, even if other aspects of the environment differ. They also suggest that genetic similarity may help predict the fitness of organisms in a new environment.

The genetic similarity we observed between pairs of populations that evolved with a shared selection pressure suggests a degree of evolutionary predictability—organisms evolved in an environment with a particular selection pressure are likely to share some mutations that are adaptive in other environments that share that selection pressure. Mutations such as those affecting the bacterioferritin, *rpfR*, and *wspE* genes were beneficial across pairs of environments with shared selection pressures. This genetic parallelism could have occurred because there are only a few genes underlying the traits under selection or because highly beneficial mutations are available in only a few of those genes (Yeaman et al. 2018).

**Figure 5:**
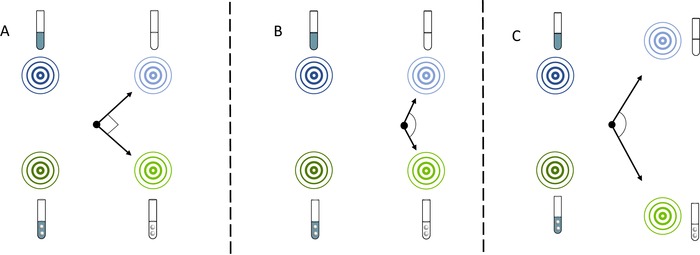
Hypothetical representations of genotypic space. Concentric circles represent the fitness peaks for each environment with thicker lines indicating higher fitness (following the assumptions in Fisher 1930). Black dots represent the ancestral genotype. Genotypic space is shown here in two dimensions for illustrative purposes, but in reality is many‐dimensional. Mutations that are beneficial in two environments are rarer when the angle between the initial genotype and the two fitness peaks is wider (Martin and Lenormand 2015). If the initial genotype were equidistant from all four fitness peaks (A), then the availability of jointly beneficial mutations would be the same between all environments that share a selection pressure. In contrast, the pattern we observed of fewer shared mutations between both low‐carbon planktonic and low‐carbon biofilm environments could occur if (B) the ancestral genotype is relatively well adapted to low‐carbon conditions or (C) the fitness peaks for low‐carbon biofilm and low‐carbon planktonic environments are relatively distant in genotypic space.

A notable exception to the pattern of similarity between environments with shared selection pressures was the low genetic similarity between populations that evolved in low‐carbon conditions (Fig. 2A). In general, low genetic similarity should occur when a small proportion of available beneficial mutations have a large fitness benefit in both environments. In a two‐dimensional landscape following Fisher's geometric model (Fig. 5), mutations that are beneficial across two environments are rare when there is a large angle between the position of the ancestral genome on the fitness landscape and the two fitness peaks (Fisher 1930; Martin and Lenormand 2015). Two nonmutually exclusive scenarios could generate a landscape in which mutations that are highly beneficial in both low‐carbon biofilm and low‐carbon planktonic environments are rare. First, the ancestral clone could be better adapted to low‐carbon environments (Fig. 5B). Second, the fitness peaks could effectively be more distant from one another in carbon‐limited environments than carbon‐replete environments (Fig. 5C).

The scenarios depicted in Figure 5B and C could both be operating in our experiment. However, several pieces of evidence suggest that the lack of similarity between biofilm and planktonic low‐carbon populations is due to the shape of the fitness landscape (Fig. 5C), rather than prior adaptation to low‐carbon conditions (Fig. 5B). First, greater genetic distance between fitness peaks in low‐carbon environments is biologically reasonable. The exopolysaccharides produced to form biofilms are carbon rich, so reproduction in low‐carbon conditions may involve a stronger trade‐off between allocating carbon to cellular growth or to biofilm production. Second, if the ancestral bacterial strain (which was originally isolated from an onion field; LiPuma et al. 2002) was better adapted to low‐carbon conditions, then we would expect smaller fitness gains in the low‐carbon environments. However, there was no difference in fitness gain between high‐ and low‐carbon environments (Fig. S1), suggesting that the scenario in Figure 5B is less likely.

Given that genetic similarity was higher in environments with shared selection pressures, we next considered if organisms that move to a new environment would be better adapted to that environment if they were more genetically similar to the native evolved populations. We showed that genetic similarity was correlated with higher fitness in all five evolutionary environments. Interestingly, in most cases all evolved populations were more fit than the ancestor, regardless of their evolutionary environment (Fig. 3). This suggests that a large fraction of the mutations that were selected in these populations were beneficial across all of the tested environments and that differences in the frequencies of mutations between environments were driven by differences in the distribution of beneficial fitness effects (Deatherage et al. 2017). For example, mutations in *rpfR* occur most frequently under biofilm conditions, but are occasionally observed in planktonic populations in this and other experiments (Traverse et al. 2013). It is likely that mutations in *rpfR* are beneficial in the planktonic environment, but either the fitness benefit is smaller or other mutations are even more beneficial under planktonic conditions and outcompete any mutations in *rpfR*. The lack of trade‐offs in adaptation to many of the environments also implies that the fitness landscapes are more complex than the simple two‐dimensional, concentric circles depicted in Figure 5.

The only trade‐off in fitness that we observe is that populations evolved under low‐carbon biofilm conditions were less fit than the ancestor in planktonic conditions. In this case, it is likely that at least some of the mutations or combinations of mutations exhibit antagonistic pleiotropy and are beneficial in the low‐carbon, biofilm environment, but detrimental in the planktonic environments. Asymmetry in trade‐offs has also been observed in studies of adaptation to different carbon sources (Travisano 1997; Lee et al. 2009). The asymmetry in fitness trade‐offs between planktonic and biofilm conditions may arise because improvements in planktonic growth can be beneficial under biofilm selection because bacteria that grow quickly in the liquid portion of the culture and then form biofilm on the bead can outcompete slowly growing strains (Lowery et al. 2017).

Understanding adaptation to environments with varying degrees of similarity is relevant in the many instances where organisms adapt to new environments, such as environmental shifts due to climate change, invasion of new species, replacement of extirpated species, and host shifts by pathogens. An important debate in restoration biology is the importance of seeking source organisms that are locally adapted (McKay et al. 2005; Weeks et al. 2011; Bucharova et al. 2017). However, our work shows that environments that appear to be similar may in fact select for dissimilar sets of adaptive mutations. Genetic similarity to existing or historical populations may be a more useful predictor of fitness in the local environment for restoration biologists when reestablishing extirpated populations or supplementing threatened populations.

### AUTHOR CONTRIBUTIONS

C.B.T. and V.S.C. conceived and designed the study. C.B.T. and C.W.M collected and analyzed data. C.B.T. wrote the manuscript, and all authors revised the manuscript.

### DATA ARCHIVING

All data and R scripts are available at Dryad https://doi.org/10.5061/dryad.53n0rf5. Raw reads submitted to NCBI SRA under BioProject number PRJNA451024 and submission number: SUB3919976. Accession numbers SAMN08956926‐SAMN08956954.

Associate Editor: K. Lythgoe

## Supporting information


**Figure S1**. Mean population size (colony forming units/transfer ± 95% confidence interval) of the ancestral strain in each environment.

**Figure S2**. Mean fitness (selection rate, per day ± 95% confidence intervals) of evolved populations in their evolutionary environment.

**Figure S3**. Mean biofilm production (± 95% confidence interval) of the ancestral clone and evolved populations.

**Figure S4**. Non‐metric multidimensional scaling (NMDS) ordination of Euclidean distance between fitness values.

**Table S1**. Sequential Bonferroni calculations (target α = 0.05) testing for significantly lower genetic similarity between treatments than within treatments for each pair of two treatments.

**Table S2**. Kendall's rank tests for correlation between fitness in an environment and genetic similarity to populations evolved in that environment.

**Table S3**.

**Table S4**.

**Text S1**. Randomization tests.
