# Supplementary material for: Parallel genetic adaptation across environments differing in mode of growth or resource availability
Source: Evol Lett. 2018 Aug 4;2(4):355–67. doi: 10.1002/evl3.75 (PMC6121802; doi:10.1002/evl3.75)
Supplement: Supplementary file 1 — Figure S1. Mean population size (colony forming units/transfer ± 95% confidence interval) of the ancestral strain in each environment. [file EVL3-2-355-s001.pdf]

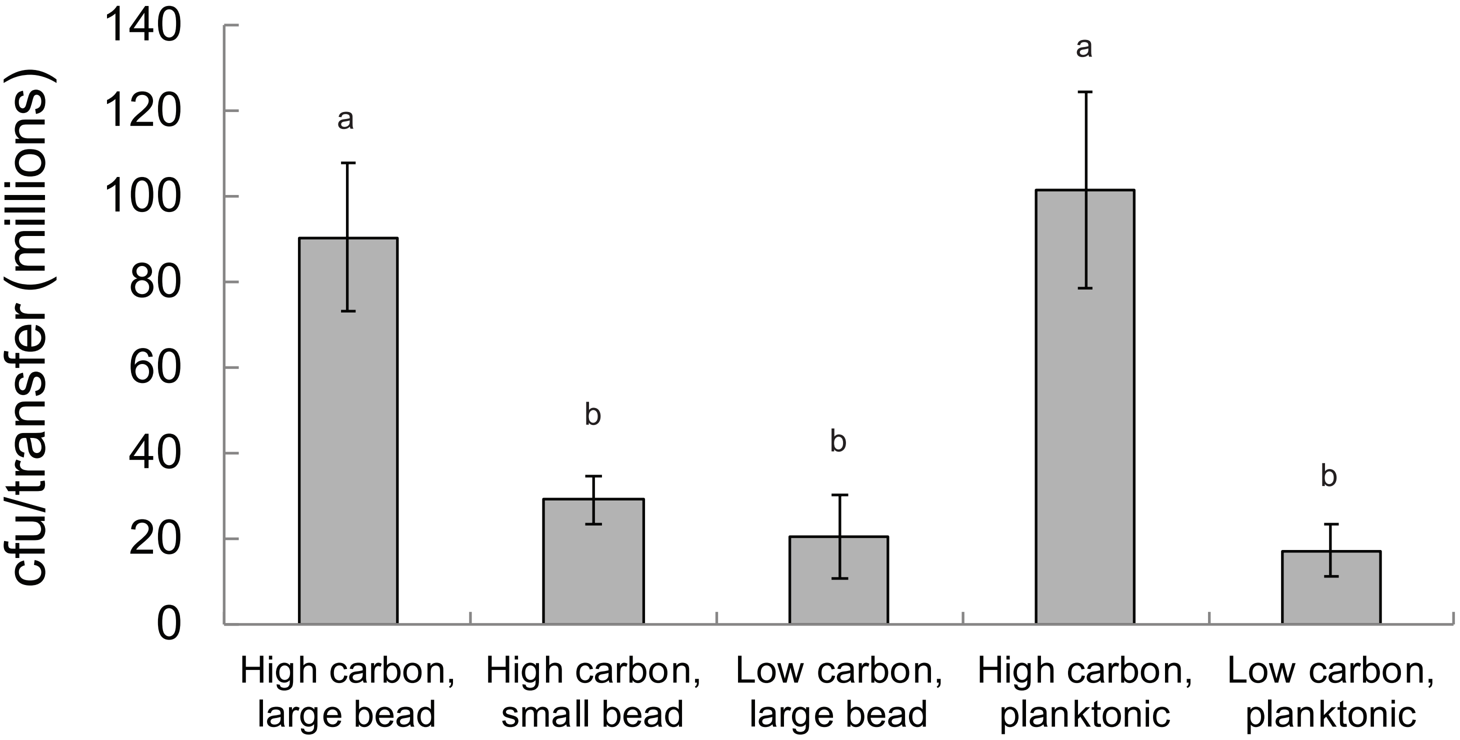

Figure S1: Mean population size (colony forming units/transfer ± 95% confidence interval) of the ancestral strain in each environment. Bars with different letters differ significantly from each other ($p < 0.05$, $n = 6$, ANOVA with Tukey's post-hoc test).
