# Supplementary material for: Parallel genetic adaptation across environments differing in mode of growth or resource availability
Source: Evol Lett. 2018 Aug 4;2(4):355–67. doi: 10.1002/evl3.75 (PMC6121802; doi:10.1002/evl3.75)
Supplement: Supplementary file 2 — Figure S2. Mean fitness (selection rate, per day ± 95% confidence intervals) of evolved populations in their evolutionary environment. [file EVL3-2-355-s002.pdf]

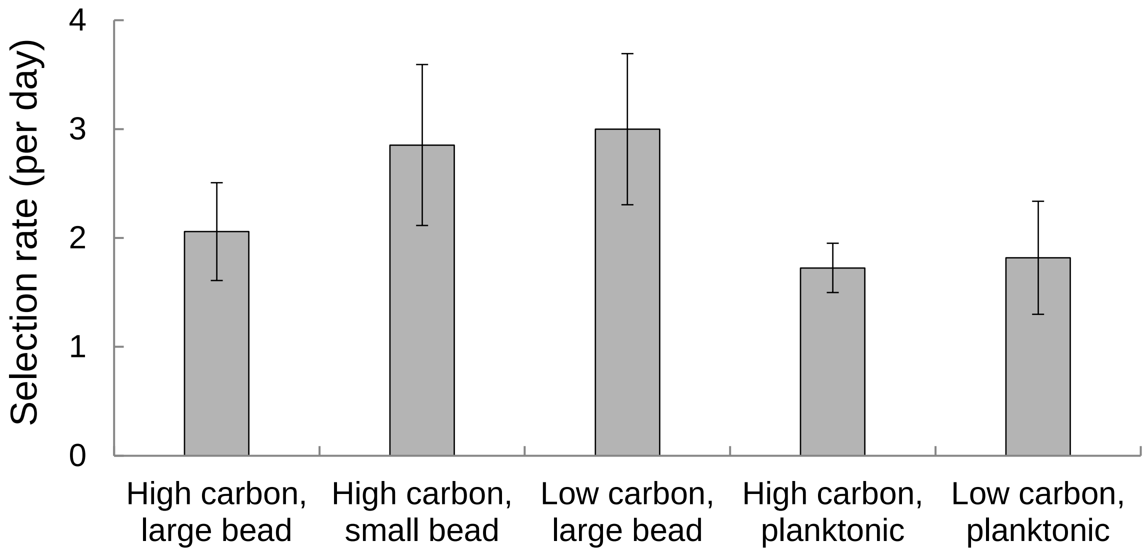

Figure S2: Mean fitness (selection rate, per day ± 95% confidence intervals) of evolved populations in their evolutionary environment. Note that these values represent independent measurements from those shown in Fig. 3.
