# Supplementary material for: Parallel genetic adaptation across environments differing in mode of growth or resource availability
Source: Evol Lett. 2018 Aug 4;2(4):355–67. doi: 10.1002/evl3.75 (PMC6121802; doi:10.1002/evl3.75)
Supplement: Supplementary file 3 — Figure S3. Mean biofilm production (± 95% confidence interval) of the ancestral clone and evolved populations. [file EVL3-2-355-s003.pdf]

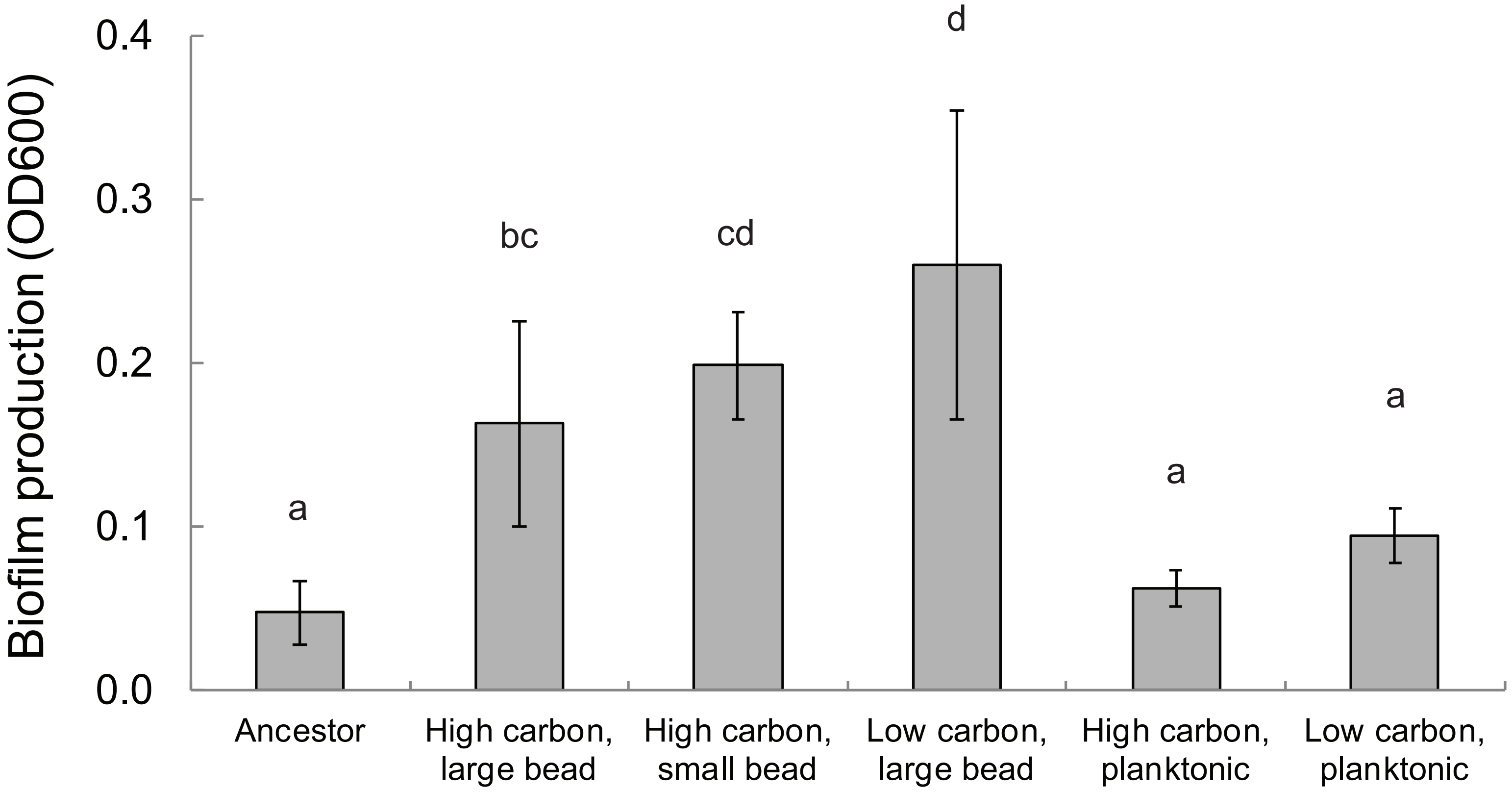

Figure S3: Mean biofilm production (± 95% confidence interval) of the ancestral clone and evolved populations. Bars with different letters differ significantly from each other ($p < 0.05$, ANOVA with Tukey's post-hoc test). Biofilm production was measured via a crystal violet assay in 96 well plates with three technical replicates per sample.
