# Supplementary material for: Parallel genetic adaptation across environments differing in mode of growth or resource availability
Source: Evol Lett. 2018 Aug 4;2(4):355–67. doi: 10.1002/evl3.75 (PMC6121802; doi:10.1002/evl3.75)
Supplement: Supplementary file 4 — Figure S4. Non‐metric multidimensional scaling (NMDS) ordination of Euclidean distance between fitness values. [file EVL3-2-355-s004.pdf]

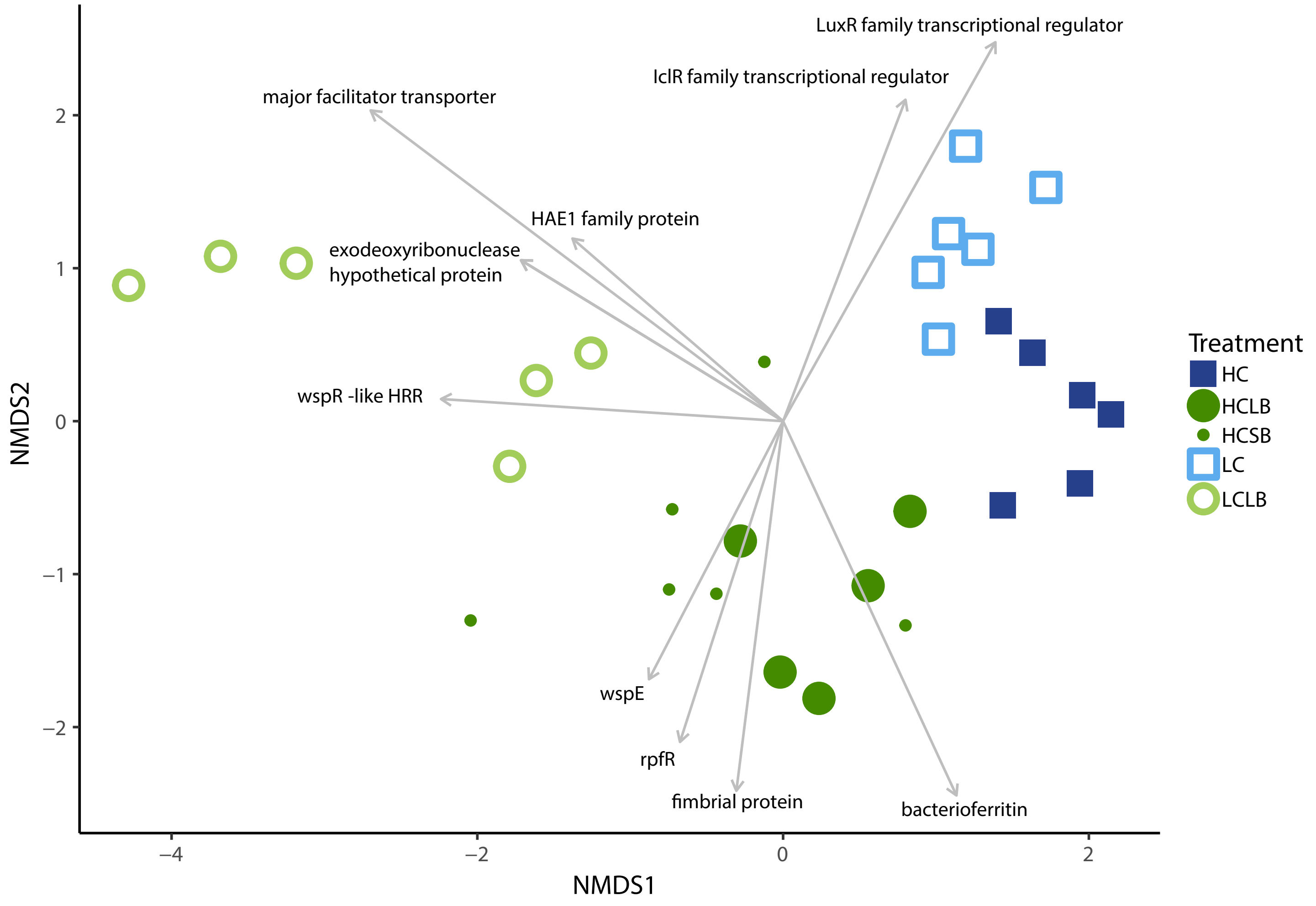

Figure S4: Non-metric multidimensional scaling (NMDS) ordination of Euclidean distance between fitness values. The vectors represent significantly correlated gene frequencies ($p<0.05$). The direction of the vector is the increasing frequency and occurrence of the gene and the length of the vector is proportional to the correlation between gene and ordination value (fitness). Calculations for ordination, distance, and vector fitting were performed in R v 3.4.4 with the vegan library v2.5-1.
