# Supplementary material for: Parallel genetic adaptation across environments differing in mode of growth or resource availability
Source: Evol Lett. 2018 Aug 4;2(4):355–67. doi: 10.1002/evl3.75 (PMC6121802; doi:10.1002/evl3.75)
Supplement: Supplementary file 5 — Table S1. Sequential Bonferroni calculations (target α = 0.05) testing for significantly lower genetic similarity between treatments than within treatments for each pair of two treatments. [file EVL3-2-355-s005.docx]

**Table S1**: Sequential Bonferroni calculations (target α = 0.05) testing for significantly lower genetic similarity between treatments than within treatments for each pair of two treatments.

| **Treatment 1** | **Treatment 2** | ***p*-value** | **rank** | **Adjusted *α*** | **Significant?** |
| --- | --- | --- | --- | --- | --- |
| Large bead, high carbon | Large bead, low carbon | 0.0022 | 1 | 0.0050 | Y |
| Small bead, high carbon | Large bead, low carbon | 0.0022 | 2 | 0.0056 | Y |
| Small bead, high carbon | Planktonic, low carbon | 0.0022 | 3 | 0.0063 | Y |
| Large bead, low carbon | Planktonic, high carbon | 0.0022 | 4 | 0.0071 | Y |
| Large bead, low carbon | Planktonic, low carbon | 0.0022 | 5 | 0.0083 | Y |
| Planktonic, high carbon | Planktonic, low carbon | 0.0022 | 6 | 0.0100 | Y |
| Large bead, high carbon | Planktonic, low carbon | 0.0043 | 7 | 0.0125 | Y |
| Small bead, high carbon | Planktonic, high carbon | 0.0043 | 8 | 0.0167 | Y |
| Large bead, high carbon | Planktonic, high carbon | 0.0433 | 9 | 0.0250 | N |
| Large bead, high carbon | Small bead, high carbon | 0.3203 | 10 | 0.0500 | N |
