# Supplementary material for: Parallel genetic adaptation across environments differing in mode of growth or resource availability
Source: Evol Lett. 2018 Aug 4;2(4):355–67. doi: 10.1002/evl3.75 (PMC6121802; doi:10.1002/evl3.75)
Supplement: Supplementary file 6 — Table S2. Kendall's rank tests for correlation between fitness in an environment and genetic similarity to populations evolved in that environment. [file EVL3-2-355-s006.docx]

**Table S2:** Kendall’s rank tests for correlation between fitness in an environment and genetic similarity to populations evolved in that environment. Correlations were calculated from data shown in Fig 3. Two tests were done for each environment- one test that included all populations and one that excluded those populations that had evolved in that environment. In all cases, the correlation was significantly positive.

|  | **All populations** | | **Only non-native populations** | |
| --- | --- | --- | --- | --- |
| **Environment** | **τ** | ***p*-value** | **τ** | ***p*-value** |
| Large bead, high carbon | 0.58097 | 0.00001 | 0.57196 | 0.00010 |
| Small bead, high carbon | 0.47644 | 0.00031 | 0.39603 | 0.00873 |
| Large bead, low carbon | 0.47436 | 0.00037 | 0.48088 | 0.00165 |
| Planktonic, high carbon | 0.49754 | 0.00015 | 0.43874 | 0.00337 |
| Planktonic, low carbon | 0.46798 | 0.00037 | 0.58103 | 0.00010 |
