# Supplementary material for: Parallel genetic adaptation across environments differing in mode of growth or resource availability
Source: Evol Lett. 2018 Aug 4;2(4):355–67. doi: 10.1002/evl3.75 (PMC6121802; doi:10.1002/evl3.75)
Supplement: Supplementary file 9 — Text S1. Randomization tests. [file EVL3-2-355-s009.pdf]

Randomization tests

We first tested whether the difference between the overall mean within-treatment similarity and the mean between-treatment similarity ($BC_{within} – BC_{between}$) was significantly greater than expected by chance. We did this by randomizing the treatment label assigned to each population while keeping the identity and frequency of mutations in a given population unchanged. For each of 10^5 randomizations, we calculated $BC_{within} – BC_{between}$ and then compared our measured $BC_{within} – BC_{between}$ to the randomized distribution. We followed a similar approach to test whether individual pairs of treatments were more genetically similar within than between treatments. Because there are only 924 possible ways to assign 12 populations to two treatments with 6 occurrences of each treatment, we used a permutation test rather than a randomization test for these comparisons. We compared the measured $BC_{within} – BC_{between}$ for each pair of treatments with $BC_{within} – BC_{between}$ for all possible permutations of treatments. To determine significance, we used a sequential Bonferroni correction (Holm 1979) with a significance level of $\alpha < 0.05$.

We also calculated the difference between the mean Bray-Curtis similarity between treatments that shared an environmental trait (BC_{shared}) and the mean Bray-Curtis similarity between treatments that did not share an environmental trait ($BC_{distinct}$). We then tested whether this difference was greater than expected by chance by randomizing the treatment assigned to each population, calculating BC_{shared} – $BC_{distinct}$ for each of 10^5 randomizations, and calculating the frequency at which the randomized distribution of $BC_{shared} – BC_{distinct}$ was as large or larger than the measured value of $BC_{shared} – BC_{distinct}$.
